# Evaluation of Performance Indicators for Malaria Control in Kinshasa from 2020 to 2023, the Democratic Republic of the Congo

**DOI:** 10.3390/epidemiologia7020055

**Published:** 2026-04-16

**Authors:** Bienvenu Bampenga Lutumbu, Kennedy Makola Mbanzulu, Germain Kieng Kapour, Madone Mandina Ndona, Josué Zanga, Jean Pierre Kambala Mukendi, Harry Kayembe, Andy Mbangama, Roger Wumba

**Affiliations:** 1 Department of Tropical Medicine, Infectious and Parasitic Diseases, University of Kinshasa, Kinshasa B.P. 127 Kinshasa XI, Democratic Republic of the Congo; lutumbubienvenu@gmail.com (B.B.L.); rogerwumba@gmail.com (R.W.); 2 Department of the Environment, School of Public Health, University of Kinshasa, Kinshasa B.P. 11850 Kinshasa I, Democratic Republic of the Congo; 3 One Health Institute for Africa, University of Kinshasa, Kinshasa B.P. 127 Kinshasa XI, Democratic Republic of the Congo; 4 Department of Internal Medicine, University of Kinshasa, Kinshasa B.P. 127 Kinshasa XI, Democratic Republic of the Congo; 5 Department of Basic Science, University of Kinshasa, Kinshasa B.P. 127 Kinshasa XI, Democratic Republic of the Congo; 6 National Reference Center for Malaria (NRCM), Bichat Claude Bernard Hospital, 75018 Paris, France

**Keywords:** malaria, performance indicators, health zone, Kinshasa, the Democratic Republic of the Congo

## Abstract

Background: In 2018, malaria remained a leading cause of morbidity and mortality in the Democratic Republic of the Congo, accounting for 44% of all outpatient visits and 22% of deaths. This led to the development of the strategic plan for 2020–2023. To meet the objectives of this renewed plan, a monitoring and evaluation program focusing on performance indicators was established. This study aimed to assess the malaria control performance indicators in Kinshasa. Methods: A descriptive cross-sectional study used the National Malaria Control Program dataset of the period 2020–2023 to analyze malaria data from 23 HZ (Health Zone) in Kinshasa. Diagnostic, therapeutic, and preventive use of LLINs (long-lasting insecticidal nets) and sulfadoxine–pyrimethamin-based IPT (intermittent preventive treatment among pregnant women) indicators were evaluated following the targeted thresholds established in the strategic plan for 2020–2023. Results: Malaria was present in all studied HZ from 2020 to 2023, with a heterogeneous distribution. The malaria incidence during the study period was 30%, with an upward trend in both suspected and confirmed cases, peaking in 2022 and showing no further fluctuations thereafter. The proportion of LLINs distributed to pregnant women during antenatal care visits was 62%, 61%, 45%, and 88% in 2020, 2021, 2022, and 2023, respectively. A total of 83.1% of suspected malaria cases were diagnosed using RDT (Rapid Diagnosis Test), and confirmed malaria cases received antimalarial treatment. Conclusions: The objectives of the 2020–2023 strategic plan were only partially achieved, and no HZ reached 100% diagnosis by RDT, with only four HZs reaching at least 95% of the target. Thirty-four HZs were able to benefit from 95% treatment with antimalarial drugs.

## 1. Introduction

Malaria is a potentially deadly disease transmitted to humans through bites from specific types of mosquitoes (female *Anopheles* species). Among the five *Plasmodium* species responsible for malaria in humans, *P. falciparum* and *P. vivax* are the most dangerous. *P. falciparum* is the parasite that causes most deaths and is also the most widespread on the African continent, leading to the highest number of deaths [1,2].

The latest WHO Malaria Report (2023) indicates that the estimated number of new malaria cases reached 263 million in 83 countries worldwide, up from 252 million in 2022 and 226 million in 2015. Malaria incidence, which is related to population growth, increased during 2015–2023 from 58 to 60.4 cases per 1000 people at risk of malaria. The total number of malaria deaths worldwide is projected to reach 597,000 in 2023, compared to 578,000 in 2015. These figures show that we are still far from meeting the targets set by WHO in its strategic plan, which called for a 90% reduction in malaria incidence and mortality rates between 2016 and 2030 relative to 2015 levels, and the elimination of malaria in 35 new countries without causing resurgence in those where it has already been eliminated [2,3,4].

In 2023, over half of malaria deaths were recorded in four countries: Nigeria (30.9%), the Democratic Republic of the Congo (DRC) (11.3%), Niger (5.9%), and the United Republic of Tanzania (4.3%) [1]. The DRC has the second-highest number of malaria cases and deaths in Africa, after Nigeria. In 2020, 12% of malaria cases and 13.2% of malaria deaths occurred in the DRC, and the country accounted for 53.1% of malaria cases in Central Africa that same year [1].

In 2018, malaria was among the leading causes of morbidity and mortality in the DRC, accounting for 44% of all outpatient visits and 22% of deaths. Approximately 97% of the population lives in areas where malaria transmission is stable for 8 to 12 months of the year. The highest levels of transmission are observed in the northern and central parts of the country. Between 2017 and 2020, the burden of malaria cases increased by 5.2%, from 308 to 324 per 1000 at-risk populations [5,6].

To assess the effectiveness of the National Malaria Control Program (NMCP) results as part of the implementation of the 2016–2020 strategic communication plan, corrected for 2020–2023, a surveillance, monitoring, evaluation, and research framework was established through conduct of several types of periodic studies and surveys, including: (1) a Demographic and Health Survey (DHS) and (2) studies and surveys focused on the indicators of malaria burden [7,8].

In the DRC, multidisciplinary strategies in the fight against malaria and basic result indicators are reported in the form of performance indicators. It includes the use of insecticide-treated mosquito nets (ITNs), intermittent preventive treatment (IPT) in pregnant women, RDT, management of all cases of probable and/or confirmed malaria, and advocacy and social mobilization for the allocation of resources necessary for the fight against malaria [8,9]. In 2024, they also included the results of the use of the RTS, S, and R21 vaccines. The present study aimed to evaluate the performance indicators of malaria control in 23 of the 35 Health Zones (HZs) in the city of Kinshasa. To better understand the trends in the performance of malaria control indicators in several HZs can assist the NMCP in adapting the intervention strategy, with important implications for designing future strategic planning.

## 2. Materials and Methods

### 2.1. Study Design, Period, and Sites

A descriptive cross-sectional study was conducted in Kinshasa to evaluate the performance indicators of malaria control. The data collected through the District Health Information Software 2 v41.8.0 (DHIS2) from 2020 to 2023 were utilized for this purpose. A total of 23 out of 35 Health Zones (HZs) of Kinshasa were involved in the current study. The studied indicators included the use of long-lasting insecticide-treated nets (LLIN), the level of use of intermittent preventive treatment (IPT) for malaria prevention among pregnant women, and the level of use of diagnostic and curative antimalarial care. To evaluate the performance of these indicators, the calculated values for each were compared to the targeted values set in the 2016–2020 MNMCP strategic plan, as adjusted for the 2020–2023 period. The targeted thresholds were 60% and 80%, respectively, for IPT and other indicators.

### 2.2. Description of the Study Framework and DHIS2 Data Collection Sites

**Figure 1:**
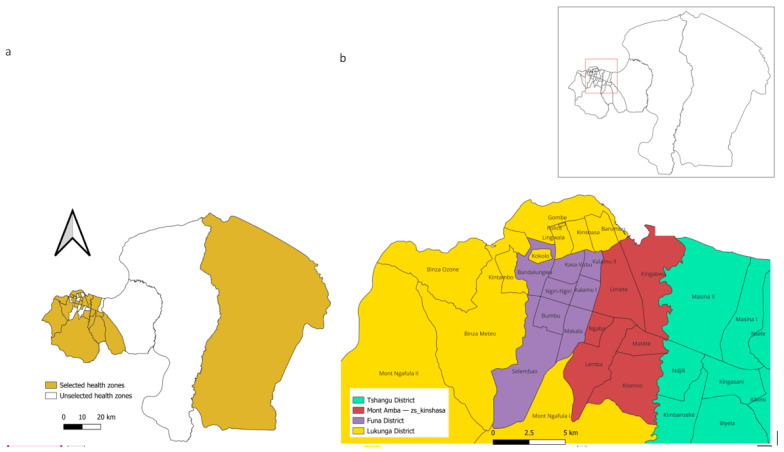
(**a**) Selected and unselected collection sites and (**b**) map of the city of Kinshasa. Production by QGIS software is open-source software.

The city of Kinshasa, the capital of the DRC, covers an area of 9965 km^2^. It remains one of the most populous cities in Africa, with an estimated population of over 17 million as of 2021 [10]. According to the health administration policy, the Regional Health Division (RHD) of Kinshasa is divided into four administrative districts (Funa, Lukunga, Mont Amba, and Tshangu), which include a total of 35 HZ as shown in Figure 1.

### 2.3. Data Collection

With the assistance of the HZ data manager, the data analyzed in this study were extracted from DHIS2. These data are routinely generated from health care activities at the Health Care Facility (HCF), where they are initially recorded using basic tools (consultation registry, prenatal consultation registry for antenatal care, and others. The data are then transcribed into National Health Information System (NHIS) forms and sent to the HZ office for analysis and validation before being encoded into DHIS2. The encoded data are then uploaded to the RHD for analysis and validation during quarterly, semiannual, and annual reviews. To ensure data security, software is locked to limit access to and manipulation of validated data.

The procedure for selecting the study HZ in Kinshasa RHD was as follows: First, a list of HZs was drawn up within each administrative district. Second, the names of these HZs were written on slips of paper, which were placed in a box. Third, two-thirds of the 35 HZs were randomly selected for inclusion in the study (Table 1).

The data downloaded included: LLIN-antenatal care visit 1, LLIN-antenatal care visit 2, LLIN- SP first dose of sulfadoxine–pyrimethamine (SP1), SP second dose (SP2), SP third dose (SP3), SP fourth dose (SP4), and data for both two age groups—<5 years old and ≥5 years old—suspected malaria cases, severe malaria cases, treated severe malaria cases, confirmed uncomplicated malaria, confirmed and treated uncomplicated malaria cases, rapid diagnostic test (RDT) performed, RDT returned positive for malaria, stained thick blood smear performed, and stained thick blood smear returned positive for malaria.

### 2.4. Descriptive and Statistical Data Analysis

The data downloaded from DHIS 2 were organized and analyzed using an Excel spreadsheet. Tables and graphs were used to present the proportions of different performances of malaria control indicators in the HZs. A map was created using QGIS 3.34 software to show the spatial distribution of malaria in Kinshasa. Malaria prevalence was calculated based on the total number of confirmed cases in selected HZs over the total population for the four years, and 95% confidence intervals were used to determine the differences between the years.

Descriptive and statistical analyses are presented in Table 2, based on definitions of parameterized variables.

**Ethical considerations**

Ethical approval of the study protocol was obtained from the National Health Ethics Committee (approval number 589/CNES/BN/PMMF/2024 date of approval 15 April 2022) in the Democratic Republic of Congo. The authorization to conduct the study was granted by the HZ authorities. The current study was carried out in collaboration with the HZ data manager, in strict compliance with the Declaration of Helsinki. The anonymized data were extracted from DHIS2. These data were from routine surveillance data collected at health facilities and stored securely, accessible only to authorized personnel. No data changes were performed to the primary data received.

## 3. Results

### 3.1. Malaria Incidence from 2020 to 2023 in Kinshasa

Overall, malaria cases increased from 2020 to 2022 (22%, 27%, and 35%, respectively), then decreased slightly in 2023 (34%) (Table 3).

**Figure 2:**
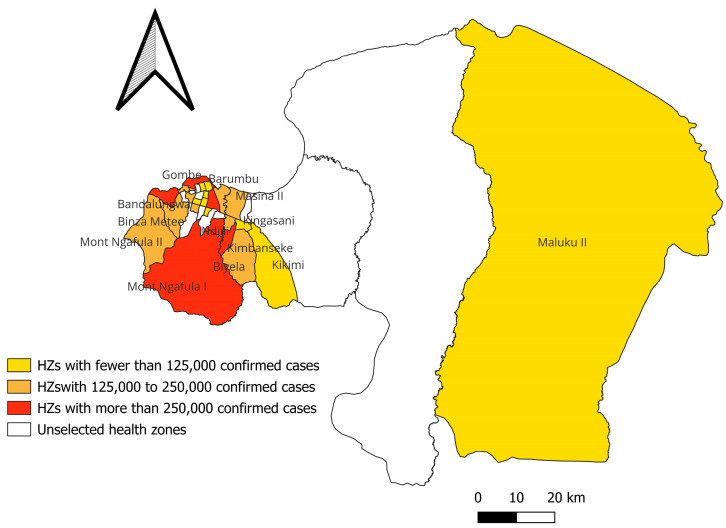
Mapping of malaria incidence in Kinshasa from 2020 to 2023; production by QGIS 3.44 software is open-source software.

Based on malaria incidence, selected HZs were stratified as follows: (1) In green, HZs with less than 100,000 cases; (2) in yellow, HZs with 100,000 to 200,000 cases; (3) in orange, HZs with 200,001 to 300,000 cases; and (4) in red, HZs with more than 300,000 cases. Non-selected HZs were mapped in white (Figure 2).

Among the studied HZ, Kimbanseke (433,326 cases), Binza Ozone (346,837 cases), Kisenso (391,503 cases), and Mont Ngafula 1 (312,604 cases) recorded the highest number of malaria cases, while Limete (311,393 cases), Masina 2 (200,705 cases), Mont Ngafula 2 (201,364 cases), and Binza Meteo (177,978 cases) are in the average range, and Kinshasa (55,507 cases), Maluku 2 (56,156 cases), and Kalamu 1 (35,066 cases) recorded the lowest number of cases, with fewer than 60,000.

### 3.2. Malaria Performance Indicators Among Pregnant Women and Children Under Five Years Old in Kinshasa from 2020 to 2023

1. Distribution of LLINs in pregnant women versus in children < 1 year old in Kinshasa from 2020 to 2023

**Figure 3:**
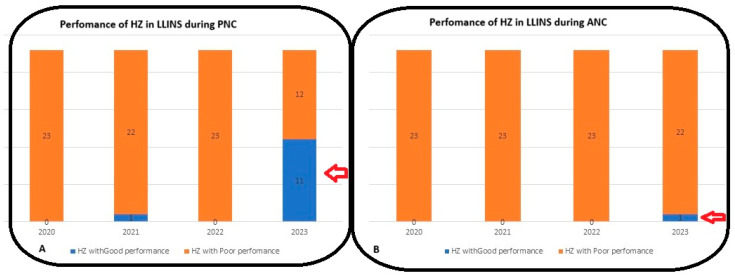
(**A**). The distribution performance of LLINs among pregnant women in 11 out of 12 HZs and (**B**) in children under one year old was only in 1 out of 12 HZs of the city of Kinshasa in 2023.

As shown in Figure 3B, in 2023, 11 out of 12 HZs (compared to only one in 2021) were effective in distributing LLINs to pregnant women, unlike the same situation in children under one year old (Figure 3A), where only Binza Meteo HZs reached 91%. The distribution of LLINs in pregnant women at ANC is reported in Table 4.

2. HZs that carried out a malaria diagnostic test according to WHO recommendations

**Figure 4:**
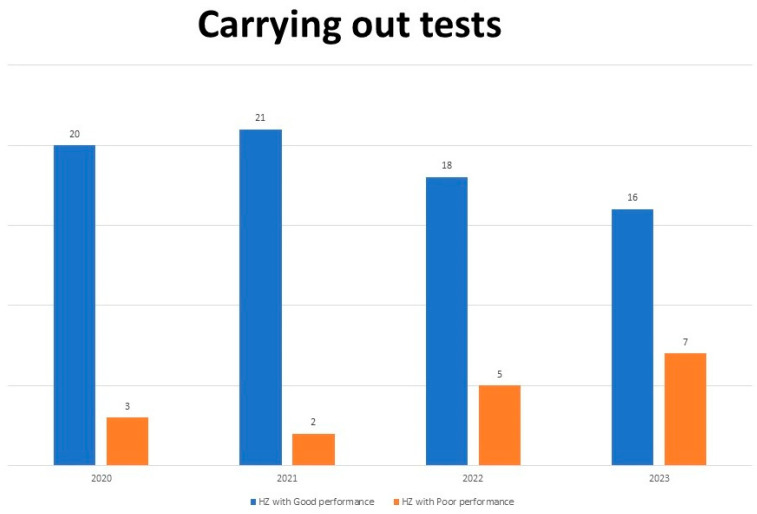
Distribution of HZs and their performance according to the performance of malaria diagnostic tests in Health Care Facilities from 2020 to 2023.

Figure 4 below shows that most HZs performed poorly from 2020 to 2023. In 2020, 87% of HZs had poor performance, and this trend continued in 2021, with 21 HZs. However, a gradual improvement emerged from 2022, with the number of HZs with good performance increasing from 22 to 30%.

Contrary to the poor performance of RDTs and stained thick blood smear in HCFs (see Figure 4 and comments), HCFs achieved 100% of the target of more than 90% diagnosis of suspected malaria cases with fever (see Table 5).

3. Malaria Treatment Performance Indicators

Malaria Cases Treated in HCFs According to the National Policy

**Figure 5:**
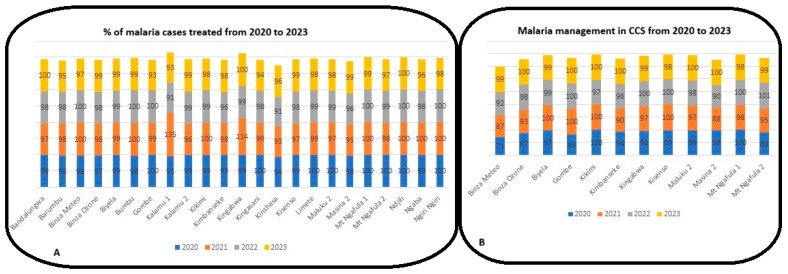
(**A**) Malaria management at the HCF level and (**B**) malaria management at the CHC level.

As shown in Figure 5A,B, while overall, no HZs reached the target, individually, only N’djili HZs consistently reached the target of 100% for patients treated for malaria cases between 2020 and 2023. Mont Ngafula 1 and Ngiri Ngiri HZs consistently reached the 100% target only between 2020 and 2022. A DHIS2 data processing error indicates that in 2021, 114% of people were treated in Kingabwa HZ.

Related to the management of malaria cases, overall, no HCF in the studied HZs consistently reached the target according to the respective standards and recommendations of NMPC and WHO between 2020 and 2023. However, some HZs, such as Kikimi, Gombe, Kisenso, Mont Ngafula 1, and Mont Ngafula 2, reached the target, but not consistently (Figure 5).

4. Intermittent Preventive Treatment (IPT) in Pregnant Women from 2020 to 2023

**Figure 6:**
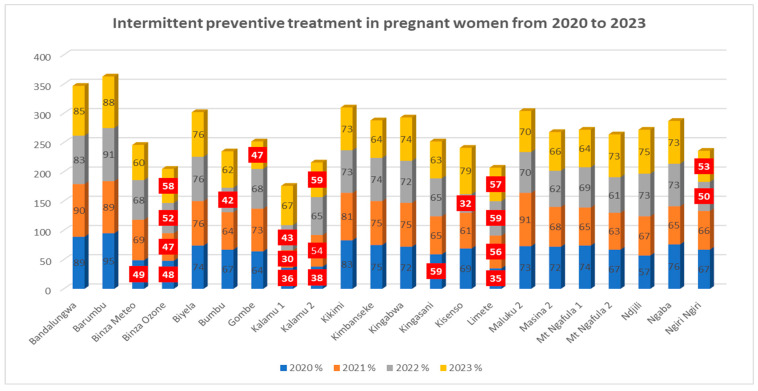
IPT in pregnant women from 2020 to 2023. The figures in red indicate the HZs that did not reach the target distribution of Sulfadoxine Pyrimethamine, which is 60%.

A few HZs did not consistently achieve the target of 60% for the use of IPT with SP during ANC2+ from 2020 to 2023 (Figure 6).

## 4. Discussion

The findings of this study reveal that, despite multiple and different measures and strategies implemented to meet the WHO objective of reducing the incidence of malaria to 30% by 2030, this incidence remained high in Kinsahsa between 2020 and 2023. The various performance indicators evaluated did not yield satisfactory results from 2020 to 2023. Aside from some biases in data collection, the findings of this study are of paramount importance.

One of the specific objectives of this study was to map the distribution of malaria in the 23 selected HZs during the study period. It emerged that some HZs, such as Kimbanseke, Mont Ngafula, Kisenso, Binza Ozone, and Kingabwa (located in the eastern, central-southern, and western parts of Kinshasa, respectively), reported more than 300,000 cases compared to the remaining assessed HZs, such as Maluku II, where prevalence was relatively low. This observation is similar to the representative and standardized mapping proposed in 2016 from a study on the risk of malaria in children aged 6 to 59 months across all HZs in Kinshasa [11]. Spatial heterogeneity of malaria incidence observed in Kinshasa is also consistent with findings from other major African cities. A study conducted in Douala, Cameroon, revealed that densely populated peripheral neighborhoods had significantly higher malaria incidence rates than central neighborhoods, primarily due to unplanned urbanization and inadequate sanitation [12,13]. Moreover, a meta-analysis conducted in several endemic countries showed that, even in settings with a high malaria burden, incidence can vary markedly depending on local factors such as altitude, access to health care, and personal protective behaviors [14]. The situation observed in Maluku II HZ, characterized by a lower incidence, can be compared to rural or semi-rural areas in other African contexts. The lower population density and distance from vector habitats could reduce the risk of malaria transmission, as demonstrated in a study conducted in rural areas of Tanzania [15].

The overall prevalence was 30%. This is consistent with national data [7] and those of some sub-Saharan African countries, as demonstrated by the 2018 DHS in Mali [16]. The prevalence increased from 22% in 2020 to 34% in 2023, despite the NMCP’s efforts in prevention, diagnosis, and treatment. Although not statistically significant (*p* < 0.05), this upward trend is concerning and could be attributed to several factors, including the rapid and often unplanned urbanization of Kinshasa, which favors the persistence of environmental conditions conducive to the proliferation of malaria vectors. In addition, possible resistance to insecticides or antimalarials could also contribute to this evolution.

Despite some occasional improvements, such as in 2021, when the Gombe HZ (94%) surpassed the target threshold of 90% set for the distribution of LLINs among pregnant women during ANC, between 2020 and 2022, no HZ reached the target. This finding is comparable to 93.1% achieved in Mali [17] in 2022, but remains slightly higher than 85% reported by the 2018 DHS in the same country [16]. However, in 2023, a notable change was recorded, with nearly half of the HZs (11 out of 23) reaching or exceeding the target.

However, the persistence of several HZs below the target in 2023 indicates that substantial intra-urban disparities still exist in Kinshasa. This situation is not unique, as a study conducted in Nigeria showed that LLIN coverage among pregnant women remained below 70% in several states, despite national campaigns, with notable disparities between rural and urban areas [18].

Despite overall improvement, some areas in Nigeria continued to underperform due to socioeconomic and logistical challenges. A study from Cameroon reports that the use of mosquito nets, even when distributed, remains limited due to cultural beliefs and climatic influences, particularly high temperatures [19].

Regarding the distribution of LLINs to children under one year of age during the preschool health examination in Kinshasa’s Health Zones, our findings revealed overall lower performance, with the averages of 41%, 33%, 31%, and 68% over the study period. Average coverage was well below targets. Only the Binza Météo HZ was considered effective in 2023, with an isolated coverage rate of 91%. These findings highlight significant gaps between the objectives set by the NMPC/DRC and reality in the field.

The fact that only one HZ was able to achieve the 90% LLIN coverage requirement among children despite four years of monitoring demonstrates that challenges persist in distributing this vector control tool. Our findings overlap with those of the previous study carried out in Kinshasa, which highlighted that LLIN distribution to young children is generally hampered by logistical challenges and the absence of structured community reminders [20].

A 2020 study in Benin found that the mosquito net coverage rate among children under five had declined significantly, particularly in urban areas [21]. Similar results were observed in Ghana, where only a few urban districts with strong NGO support achieved acceptable coverage rates, while the majority remained below target thresholds [22].

The WHO Report 2023 stated that only 64% of at-risk children under five in endemic countries sleep under an LLIN [1]. However, these figures contrast with those reported in Côte d’Ivoire by Konan and colleagues in 2019, who found very satisfactory rates [23].

At the HCF level, the majority of patients diagnosed with malaria were treated according to national guideline policy, with a treatment rate ranging between 95% and 100% during the study period. Some HZs, such as Gombe, Kingasani, Ndjili, and Ngiri-Ngiri, maintained 100% treatment for several years. However, some data anomalies were noted, notably rates above 100% in Kalamu 1 and Kingabwa, likely due to data entry errors in DHIS2 (health data management tool). These observations are similar to those made in Senegal [24]. However, they far exceed those reported in Côte d’Ivoire [25].

The information collected in the CHCs also showed satisfactory progress. In 2020, only Kikimi and Mont Ngafula 1 achieved a 100% treatment rate. By 2021, other malaria treatment centers, such as Biyela, Gombe, and Kisenso, had reached this threshold. By 2023, all malaria treatment centers recorded treatment rates ranging between 99% and 100%.

These observations highlight strong compliance with national malaria treatment policies, both at the HCF and CHC levels of Kinshasa, compared with data recorded at national and regional levels. In 2022, the NMCP/DRC reported that only 53% of malaria cases were treated according to national protocol nationwide, across all facilities [26].

In Kinshasa, this poor practice is more commonly observed in urban hospitals and clinics, where the use of inappropriate medications or medications that do not comply with national policy is noted. According to the World Health Organization’s 2023 report, data from the WHO African Region indicate that only 60–70% of diagnosed cases are treated correctly following the protocols [1]. A pooled analysis of clinical trials across East Africa, including Uganda, Kenya, and Tanzania, demonstrates that provider adherence to artemisinin-based combination therapy (ACT) protocols remains inconsistent, with rates typically ranging between 55% and 80% [27]. This variability is largely driven by clinicians’ propensity to prescribe antimalarials despite negative diagnostic test results and fluctuating availability of rapid diagnostic tests (RDTs) [27].

Regarding sulfadoxine–pyrimethamine (SP)-based intermittent preventive treatment (IPT) among pregnant women in Kinshasa between 2020 and 2023, results highlighted very heterogeneous coverage rates across HZs. Some HZs, such as Bandalungwa, Barumbu, Kikimi, Biyela, and Kingabwa, showed satisfactory rates (70–90%) throughout the period. Others, such as Binza Ozone, Kalamu 1, and Limete, had low rates (often below 60%). It is noted that in some areas, the IPT distribution rate exceeded 100%, likely due to inadequate data entry or improper handling of figures.

Overall, Kinshasa’s performance was below the targets set by the WHO and the NMCP (target of ≥80% coverage). These findings align with broader national data from the Democratic Republic of Congo, which consistently report suboptimal IPTp-SP uptake across various provinces [28]. In some Kinshasa HZs, this rate was higher than 88.8% reported in Mali and well above the 40% reported in Côte d’Ivoire [23]. This poor performance is also noted in the 2023 WHO Malaria Report, where the average IPT coverage among pregnant women in Africa was around 55–60% for at least two doses, except countries such as Gambia and Senegal, which achieved rates of 80%, due to the increased integration of IPT into primary health care [20]. This poor performance in Kinshasa may be attributed to stockouts of SP, inadequate ANC follow-up, and a lack of targeted community outreach.

In our study, data were limited to malaria cases reported from Health Care Facilities and stored in DHIS2, without considering malaria cases occurring in the community that were not being reported to the HZ office. Therefore, the incidence of malaria in this study could be underestimated.

The data anomalies were observed, including coverage rates exceeding 100%, such as in Kalamu 1 and Kingabwa, which are likely attributable to data entry errors in DHIS2, and are also acknowledged as a study limitation.

## 5. Conclusions

This study highlights several trends in the performance of malaria control indicators. The observed disparities in HZs call for targeted interventions adapted to local realities. While notable progress has been made, particularly in the distribution of mosquito nets and compliance with treatment protocols, significant challenges remain in terms of preventing malaria among children, accessing quality diagnosis, and rigorously monitoring indicators. A more targeted, integrated, and community-based approach appears essential to strengthen the effectiveness of antimalarial strategies in Kinshasa and move towards sustainable control of the disease. There is also a current need to improve the report data in DHIS2 to avoid errors likely attributable to data entry in DHIS2.

## Figures and Tables

**Table 1:** Distribution of 23 selected HZs by district.

Districts	Selected HZ
Funa	Bandalungwa
	2. Bumbu
	3. Kalamu 1
	4. Kalamu 2
	5. Ngiri-Ngiri
Lukunga	6. Binza-Meteo *
	7. Binza-Ozone *
	8. Gombe *
	9. Kinshasa
	10. Mont-Ngafula 1 *
	11. Mont-Ngafula 2 *
Mont Amba	12. Kingabwa 1 *
	13. Kinsenso *
	14. Limete
	15. Ngaba
	16. Kingabwa 2
Tshangu	17. Biyela *
	18. Kikimi *
	19. Kimbanseke *
	20. Kingasani
	21. Masina 2 *
	22. Ndjili
	23. Maluku 2 *

*: Health Zones with a community health care site. The numbers 1 and 2 added next to the same name imply two different health zones.

**Table 2:** Descriptive and statistical analyses of variables.

Variables	Numerator (a)	Denominator (b)	Calculation
Percentage of LLINs distributed to pregnant women during ANC	Number of LLINs distributed to pregnant women during ANC in HZ	Number of women seen at ANC	$\frac{a}{b}\times 100$
Percentage of LLINs distributed to children under one year of age during SMC	Number of LLINs distributed to children under one year of age in health facilities	Number of children vaccinated with VAR	$\frac{a}{b}\times 100$
Percentage of suspected malaria cases tested (RDT or stained thick blood smear) in HCF	Number of suspected cases tested with RDT and/or stained thick blood smear in HCF	Number of suspected cases received in HCF	$\frac{a}{b}\times 100$
Percentage of uncomplicated malaria cases receiving antimalarial treatment according to national protocol at the health and social care centers	Number of uncomplicated malaria cases treated in health and social care centers according to national policy	Number of confirmed uncomplicated malaria cases	$\frac{a}{b}\times 100$
Percentage of suspected malaria cases who underwent RDT in HCF	Suspected malaria cases who underwent RDT in HCF	Suspected malaria cases received in HCF	$\frac{a}{b}\times 100$
Percentage of uncomplicated malaria cases receiving antimalarial treatment according to the national protocol at the HCF level	Uncomplicated malaria cases received at the HCF level who received treatment according to the national protocol	All uncomplicated malaria cases received at the HCF level	$\frac{a}{b}\times 100$
Percentage of pregnant women receiving intermittent preventive treatment (IPT)	Number of pregnant women treated with SP	Number of women receiving ANC	$\frac{a}{b}\times 100$

**Table 3:** Malaria incidence results from 2020 to 2023.

Year	Population	Malaria Cases	%	IC 95%
2021	5,831,595	1,582,272	27%	3.3–50.7
2022	6,006,543	2,098,068	35%	4.2–74.2
2023	6,186,740	2,089,208	34%	14.6–82.6

**Table 4:** Distribution of LLINs in pregnant women at ANC.

Health Zone	2020			2021			2022			2023		
	LLINs ANC	Wo ANC	% of Distributed LLINs	LLINs ANC	Wo ANC	% of Distributed LLINs	LLINs ANC	Wo ANC	% of Distributed LLINs	LLINs ANC	Wo ANC	% of Distributed LLINs
Bandalungwa	1686	3028	56	1332	3103	43	1275	3432	37	3814	3771	101
Barumbu	2008	2694	75	1672	3227	52	1630	3405	48	2634	3460	76
Binza Meteo	7009	11,448	61	7076	11,245	63	7712	12,802	60	15,133	14,958	101
Binza Ozone	4889	9093	54	7346	10,341	71	6667	12,847	52	14,588	16,132	90
Biyela	5077	7083	72	6165	8360	74	3348	11,023	30	9159	12,152	75
Bumbu	6834	9414	73	6861	11,931	58	6813	26,957	25	16,799	19,558	86
Gombe	2070	2787	74	5264	5594	94	3243	5355	61	3385	3581	95
Kalamu 1	1226	2720	45	1351	2780	49	754	3779	20	3792	5028	75
Kalamu 2	1816	2382	76	1560	2227	70	905	2493	36	2961	3393	87
Kikimi	6035	10,876	55	6232	13,268	47	6455	16,716	39	13,691	16,366	84
Kimbanseke	4515	8978	50	6523	10,059	65	7788	15,859	49	13,621	17,515	78
Kingabwa	5574	10,886	51	6087	12,887	47	5254	11,212	47	12,222	12,271	100
Kingasani	6045	10,132	60	7795	14,700	53	8842	15,892	56	11,461	12,629	91
Kinshasa	4664	7439	63	4854	7060	69	4444	8826	50	5057	7091	71
Kisenso	5311	7307	73	8806	13,549	65	8262	19,244	43	17,703	19,584	90
Limete	4560	9205	50	4068	9079	45	2893	10,376	28	9844	10,723	92
Maluku 2	2721	3710	73	2670	3657	73	2452	3617	68	2476	2214	112
Masina 2	3485	6197	56	5472	10,183	54	5891	14,062	42	11,362	13,570	84
Mont Ngafula 1	6542	11,330	58	7632	12,154	63	6767	13,858	49	14,424	15,483	93
Mont Ngafula 2	3021	4886	62	2684	4726	57	2187	6836	32	6878	8373	82
Ndjili	5558	9106	61	7170	9447	76	5758	9076	63	8940	10,128	88
Ngaba	3105	4582	68	2626	4481	59	3137	5757	54	5090	5376	95
Ngiri Ngiri	1403	2611	54	1235	2598	48	1314	3230	41	5583	6455	86
Total	95,154	157,894	62	112,481	186,656	61	103,791	236,654	45	210,617	239,811	88

Wo: women ANC: antenatal care. HZs are listed alphabetically.

**Table 5:** Proportion of suspected malaria cases diagnosed by RDT in HCFs.

Health Zone	2020			2021			2022			2023		
	FEVER	RDT	%	FEVER	RDT	%	FEVER	RDT	%	FEVER	RDT	%
Binza Meteo	7055	6620	94	10,308	10,044	97	9727	9479	97	8853	8839	100
Binza Ozone	2388	2158	90	7092	6805	96	7434	7255	98	10,976	11,122	101
Biyela	6782	6782	100	9820	9776	100	10,037	9926	99	11,353	11,348	100
Gombe	4843	4843	100	7223	7223	100	7052	7052	100	6611	6611	100
Kikimi	1102	1043	95	1520	1446	95	2698	2627	97	2088	2080	100
Kimbanseke	2250	2120	94	3158	3072	97	3480	3323	95	3214	3175	99
Kingabwa	2430	2183	90	5541	5453	98	6507	6497	100	5805	5761	99
Kisenso	2331	2201	94	3462	3489	101	5243	5067	97	6449	6435	100
Maluku 2	5446	5143	94	4363	4368	100	4314	4294	100	4476	4474	100
Masina 2	1546	1386	90	1803	1647	91	2037	1988	98	2581	2536	98
Mt Ngafula 1	5451	5402	99	6402	6287	98	9344	8725	93	9424	9400	100
Mt Ngafula 2	6672	6319	95	6187	5814	94	7343	6579	90	7333	7203	98
Total	48,296	46,200	95	66,879	65,424	97	75,216	72,812	97	79,163	78,984	100

## Data Availability

All data supporting the study findings are included in this published article and can be found in DHIS2.

## Abbreviations

The following abbreviations are used in this manuscript:

DRC	the Democratic Republic of the Congo
WHO	World Health Organization
MNCP	National Malaria Control Program
HZ	Health Zones
LLIN	Long-lasting insecticidal nets
IPT	Intermittent preventive treatment
RDT	Rapid Diagnosis Test
DHS	Demographic and Health Survey
DHIS2	District Health Information Software 2
RHD	Regional Health Division
HCF	Health Care Facility
SP	Sulfadoxine–pyrimethamine
PNC	Post-natal care
ANC	Antenatal care visit
